# Podocyte GSK3α is important for autophagy and its loss detrimental for glomerular function

**DOI:** 10.1096/fba.2019-00011

**Published:** 2019-07-09

**Authors:** Jennifer A. Hurcombe, Abigail C. Lay, Lan Ni, Alexandra F. Barrington, Jim R. Woodgett, Susan E. Quaggin, Gavin I. Welsh, Richard J. Coward

**Affiliations:** ^1^ Bristol Renal University of Bristol Bristol UK; ^2^ Lunenfeld‐Tanenbaum Research Institute Sinai Health System & University of Toronto Toronto ON Canada; ^3^ Feinberg Cardiovascular Research Institute Northwestern University Feinberg School of Medicine Chicago Illinois

**Keywords:** Adriamycin nephropathy, albuminuria, diabetic nephropathy, insulin signaling

## Abstract

Podocytes are key cells in maintaining the integrity of the glomerular filtration barrier and preventing albuminuria. Glycogen synthase kinase 3 (GSK3) is a multi‐functional serine/threonine kinase existing as two distinct but related isoforms (α and β). In the podocyte it has previously been reported that inhibition of the β isoform is beneficial in attenuating a variety of glomerular disease models but loss of both isoforms is catastrophic. However, it is not known what the role of GSK3α is in these cells. We now show that GSK3α is present and dynamically modulated in podocytes. When GSK3α is transgenically knocked down specifically in the podocytes of mice it causes mild but significant albuminuria by 6 weeks of life. Its loss also does not protect in models of diabetic or Adriamycin‐induced nephropathy. In vitro deletion of podocyte GSK3α causes cell death and impaired autophagic flux suggesting it is important for this key cellular process. Collectively this work shows that GSK3α is important for podocyte health and that augmenting its function may be beneficial in treating glomerular disease.

**Abbreviations**
AKT: protein kinase B
BSA: bovine serum albumin
ELISA: enzyme‐linked immunosorbent assay
FBS: fetal bovine serum
FSGS: focal segmental glomerulosclerosis
GSK3: glycogen synthase kinase 3
KO: knockout
MAPK: mitogen‐activated protein kinase
PAS: periodic acid Schiff
PBS: phosphate buffered saline
PCR: polymerase chain reaction
PDGF: platelet‐derived growth factor
PECAM‐1: platelet endothelial cell adhesion molecule‐1
STZ: streptozotocin
TBS‐T: tris‐buffered saline‐tween 20
uACR: urinary albumin creatinine ratio
WT: wild‐type
WT‐1: Wilm's tumor protein

## 1 INTRODUCTION

The podocyte is critical to the proper functioning of the mammalian glomerular filtration barrier and the prevention of albuminuria, a hallmark of kidney disease. Therefore, identifying signaling molecules and pathways that regulate podocyte function and which can be pharmacologically targeted is important.

Glycogen synthase kinase 3 (GSK3) is a multi‐functional serine/threonine kinase which regulates a number of distinct signaling pathways.^1^ In mammals, GSK3 exists as two separate but related isoforms (α and β) with overall homology of 85% and highly conserved kinase domains (97% homology).^2^ Because of this structural similarity, currently available GSK3 inhibitors are not isoform selective. However, there is evidence that GSK3α and β are not entirely redundant and have different functions: GSK3β null mice die during late embryogenesis as a result of impaired NFκB signaling and cardiac defects 3, 4 whereas whole body GSK3α knockout mice are viable and interestingly, exhibit enhanced insulin sensitivity in a strain‐dependent manner.^5^ Multiple cell‐specific GSK3 knockout mouse models have been described and indicate that the functions of the GSK3 isoforms are cell‐type dependent.^6^, ^7^, ^8^, ^9^

We have recently shown that GSK3 is a crucial regulator of podocyte and kidney function and that simultaneous loss of both GSK3 isoforms in podocytes is highly detrimental.^10^ Developmental deletion of GSK3 results in neonatal death associated with renal failure while knockout in mature podocytes leads to a spectrum of renal disease. We also showed that prolonged pharmacological inhibition of GSK3 in rats using lithium caused significant proteinuria with evidence of focal segmental glomerulosclerosis (FSGS).^10^ This is consistent with reports of FSGS and renal failure in patients on long‐term lithium therapy for conditions such as bipolar disorder.^11^, ^12^ In contrast, a number of recent studies suggest that partial pharmacological inhibition of GSK3 as well as specific genetic knockout of the β isoform in podocytes can be beneficial in a number of experimental glomerular disease models including diabetic nephropathy, lupus nephritis and Adriamycin nephropathy.^13^, ^14^, ^15^, ^16^, ^17^ However, the role of podocyte GSK3α has received little consideration and it is not known whether this isoform has a specific role in podocyte homeostasis or if its loss can also have a podocyte protective effect.

Given our interest in podocyte insulin signaling^18^ and the suggestion that specifically inhibiting GSK3α could enhance cellular insulin sensitivity we were keen to study the specific role of this GSK3 isoform in the podocyte. We therefore developed a mouse and podocyte cell model to do this.

We show here that reduced podocyte expression of GSK3α does not attenuate albuminuria in streptozotocin (STZ) diabetes or Adriamycin nephropathy models in mice. Moreover, near complete knockout of GSK3α in conditionally immortalized podocytes did not result in improved insulin sensitivity or attenuation of Adriamycin induced injury. Conversely, we found that loss of podocyte GSK3α is mildly detrimental and reveal a role for this isoform in maintaining podocyte autophagic flux.

## 2 MATERIALS AND METHODS

### 2.1 Murine insulin stimulation

Following an overnight fast, wild‐type mice were given an intraperitoneal injection of insulin (Novo Nordisk) at 1 unit/kg body weight (6 nmol/L). Mice were sacrificed after 10 minutes and the kidneys snap frozen in liquid nitrogen.

### 2.2 Immunofluorescence staining of kidney sections

Frozen kidneys were sectioned at 5 μm. Sections were blocked in phosphate buffered saline (PBS) containing 3% bovine serum albumin (BSA) and 0.3% triton X‐100 for 1 hour, then incubated with primary antibodies overnight at 4°C (pGSK3α 1:100; pGSK3β 1:100; nephrin 1:300; Wilm's tumor protein [WT‐1] 1:200). Following three PBS rinses, sections were incubated with fluorophore‐conjugated secondary antibodies (Life Technologies) for 1 hour at room temperature. Tissues were imaged using a Leica DM2000 microscope and micrographs taken with Leica Application Suite. Image analysis was performed with ImageJ; all images were contrast enhanced using the same parameters.

### 2.3 Insulin and Adriamycin stimulation of cultured podocytes

Conditionally immortalized mouse podocyte cell lines were used as previously described.^19^, ^20^ Cells were serum starved for 4 hours then stimulated with insulin (Novo Nordisk) at 10 nm and 100 nmol/L for 10 minutes.

For the Adriamycin injury model, cells were incubated with Adriamycin (Sigma) at the concentrations indicated for 24 hours.

### 2.4 Western blotting

Cultured cells were lysed in radioimmunoprecipitation assay buffer supplemented with protease and phosphatase inhibitors (Sigma). Ten to thirty micrograms of protein was resolved by electrophoresis then transferred to a polyvinylidene difluoride membrane (Millipore). Membranes were blocked in TRIS‐buffered saline with 0.1% tween 20 and 5% BSA for 1 hour then incubated overnight with primary antibody at a dilution of 1:1000. Membranes were washed before incubation with horseradish peroxidase conjugated secondary antibody (Sigma). Immunoreactive bands were visualized using Clarity ECL Western blotting substrate (Biorad) on a GE AI600 imager. Densitometry was performed using ImageJ software.

### 2.5 Mouse models

Mice in which exon two of GSK3α has been flanked by loxP sites^5^ were crossed with podocin Cre mice^21^ to generate podocyte‐specific GSK3α knockdown animals from embryonic day 12 (podGSK3αKD mice).

Diabetes was induced in mice at 6 weeks of age by intraperitoneal injection of STZ (Sigma) at 50 mg/kg body weight for five consecutive days. Mice injected with an identical volume of citrate buffer served as sham controls.

Adriamycin nephropathy was induced in mice at 8‐10 weeks of age by a single tail vein injection of Adriamycin (Sigma) at 12 mg/kg body weight. These were studied for 2 weeks.

For STZ and Adriamycin models, mice were backcrossed for four generations onto a DBA2J strain background. Both sexes were studied.

Transgenic mouse work was carried out in accordance with the University of Bristol's institutional guidelines and procedures approved by the United Kingdom (UK) Home Office in accordance with UK legislation.

### 2.6 Podocyte primary culture

Glomeruli were isolated from podGSK3α KD and littermate control mice following dynabead perfusion as described previously.^22^ Glomeruli were plated out in Roswell Park Memorial Institute (RPMI) 1640 media supplemented with 10% fetal bovine serum and 5% penicillin/streptomycin and incubated at 37°C. After 6 days, primary podocytes were harvested by trypsinization and cells were passed through a 40 μm cell strainer to remove glomeruli.

### 2.7 End point PCR

Mouse genotyping was performed using 5× PCRbio hs taq DNA polymerase (Insight) and the following primers:

Cre forward GTGCAACTTGAATAACCGGAAATGG
Cre reverse AGAGTCATCCTTACCGCCGTAAATCAAT
GSK3α forward CCCCCACCAAGTGATTTCACTGCTA
GSK3α reverse AACATGAAATTCCGGGCTCCAACTCTAT

RNA was isolated from podocyte primary cells using an Rneasy kit (Qiagen) and cDNA synthesized using a high capacity RNA to cDNA kit (Thermofisher Scientific). Primers used were:

WT‐1 forward GAGAGCCAGCCTACCATCC
WT‐1 reverse GGGTCCTGGTGTTTGAAGCAA
Platelet endothelial cell adhesion molecule‐1 (PECAM) forward CAAGCAAAGCAGTGA
PECAM reverse AGCAGGACAGGTCCAACAAC
Platelet‐derived growth factor (PDGF) forward ACTTCTGTTGCTACACGAAGC
PDGF reverse CGGTTGAGTCAGTGGAGTCC

### 2.8 Urinary albumin and creatinine measurements

Albumin and creatinine levels in mouse urine were measured using a mouse‐specific albumin ELISA (Bethyl) and creatinine companion kit (Exocell), following the manufacturers' methodology.

### 2.9 Periodic acid Schiff staining

Kidneys were fixed in 10% buffered neutral formalin, further processed and paraffin embedded. Three micrometer sections were cut and stained using a Periodic acid Schiff staining kit (Sigma) according to the manufacturers' instructions. Tissues were imaged using a Leica DN2000 microscope and micrographs taken using Leica Application Suite software. Image analysis was performed using ImageJ; all images were contrast enhanced using the same parameters.

Glomerulosclerosis was scored for each glomerulus as follows: 0 = normal glomeruli; 1 = up to 25% involvement; 2 = 25%‐50% involvement; 3 = 50%‐75% involvement; 4 = over 75% involvement. The glomerulosclerosis index was calculated according to the formula ([1 × N1] + [2 × N2] + [3 × N3] + [4 × N4])/(N0 + N1 + N2 + N3 + N4), where N*x* is the number of glomeruli with each given score.

### 2.10 Lentiviral transduction of a conditionally immortalized GSK3α floxed podocyte cell line

Kidneys were isolated from GSK3α^fl/fl^ mice and used to make a temperature‐sensitive SV40 conditionally immortalized podocyte cell line as described previously.^19^ Cells were cultured at 33°C and when 50% confluent were transduced with a lentivirus expressing Cre recombinase as described previously.^10^ Transduction was in RPMI media with hexadimethrine bromide (Sigma) at 4 μg/mL and the virus used at a multiplicity of infection of 1. Following a 24‐hour incubation, the lentivirus was removed and replaced with fresh media. Cells were thermo‐switched to 38°C and incubated for a further 7 days before imaging and protein extraction.

To determine ciGSK3αKO cell number, cells were washed three times in PBS, the nuclei stained with Hoechst (Sigma) at 1 μg/mL and imaged using an IN Cell analyzer 2200 (GE Healthcare) and data analyzed using IN Cell analyzer Developer software (GE Healthcare).

### 2.11 Determination of podocyte number

Immunofluorescent analysis of mouse kidney sections was carried out as described above using a WT‐1 antibody (Abcam). 4′,6‐diamidino‐2‐phenylindole (DAPI) was used to counterstain nuclei. ImageJ software was used to count WT‐1 positive nuclei and expressed as a percentage of the total number of DAPI stained cells per glomerulus.

### 2.12 Antibodies

Antibodies were obtained from Cell Signalling Technology: (pGSK3α [Ser 21] #9316; pGSK3β [Ser 9] #9323; total GSK3α/β #5676; total GSK3α #9338; pAKT [Ser 473] #4060; total AKT #4691; P‐p44/42 MAPK #4370; p44/42 MAPK #4695); Acris (Nephrin #BP5030); Sigma (β‐actin clone AC‐15; GAPDH #G8795); Merck Millipore (WT‐1 clone 6F‐H2, used for western blotting), Abcam (WT‐1 #89901) and Genetex (SV40 T antigen #134377).

### 2.13 Statistics

Statistical analysis was performed using GraphPad Prism software. When comparing two groups *t* tests were used. When comparing more than two groups analysis of variance (ANOVA) was used. Statistical tests used and n numbers are shown in figure legends. Data are presented as the mean and error bars represent the standard error of the mean. *P* < 0.05 was deemed statistically significant.

## 3 RESULTS

### 3.1 Both GSK3α and GSK3β are phosphorylated in podocytes of murine glomeruli and in murine conditionally immortalized podocytes in response to insulin

**Figure 1:**
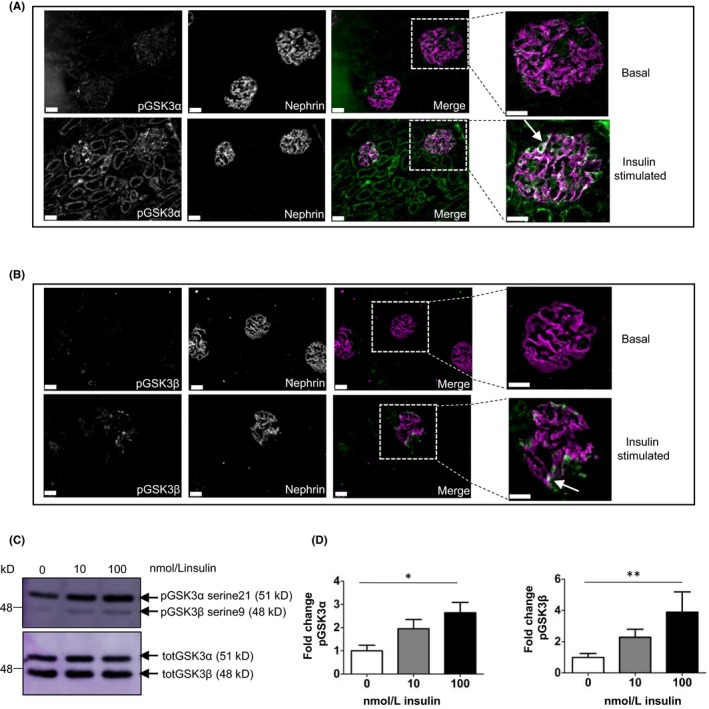
GSK3α as well as β is phosphorylated in the podocyte in the murine glomerulus and in murine conditionally immortalized podocytes. (A, B) Immunofluorescent analysis of serine 21 phosphorylated GSK3α (A) and serine 9 phosphorylation of GSK3β (B) performed in mice that were fasted overnight and then given insulin (6 nmol/L) for 10 min. Podocyte‐specific phosphorylation of GSK3 (green) is shown by co‐localization with Nephrin (magenta)(white demonstrates co‐localization) arrowed in magnified images, scale bar = 25 μm. (C, D) Insulin increases inhibitory phosphorylation of GSK3 in conditionally immortalized murine podocytes. Representative blot (C) and densitometry shown (D). Unpaired *t* test, *P < 0.05; **P < 0.01, n = 3 experiments

We initially established that both GSK3α and GSK3β were expressed and dynamically regulated in the podocyte by studying wild‐type mice challenged with insulin after an overnight fast (Figure 1A,B). This revealed the rapid phosphorylation, and hence inactivation, of GSKα, at serine 21, and GSK3β, at serine 9, in the kidney. Both isoforms were phosphorylated in glomeruli and tubules. However, within the glomerulus phosphorylated GSK3 isoforms were highly enriched in the podocyte cell fraction. This was particularly clear for GSK3α as revealed by 3D reconstructive imaging of insulin treated glomeruli (Video S1). Western blotting of conditionally immortalized podocytes confirmed these phosphorylation findings in vitro (Figure 1C,D). Preliminary dose‐response experiments showed that our mouse podocyte cell line activated the PI3K pathway in response to insulin at 1 nmol/L but this was more apparent at higher doses (Figure S1). Therefore, in subsequent experiments, cells were stimulated with insulin at 10 and 100 nmol/L. Although greater than the levels of insulin present in diabetic patients, 10‐100 nmol/L is the standard dose used in the literature to elucidate insulin driven signaling pathways.^23^, ^24^, ^25^, ^26^, ^27^, ^28^ Our in vivo studies were performed with insulin doses of 6 nmol/L.

### 3.2 Generation of podocyte‐specific GSK3α knockdown mice

**Figure 2:**
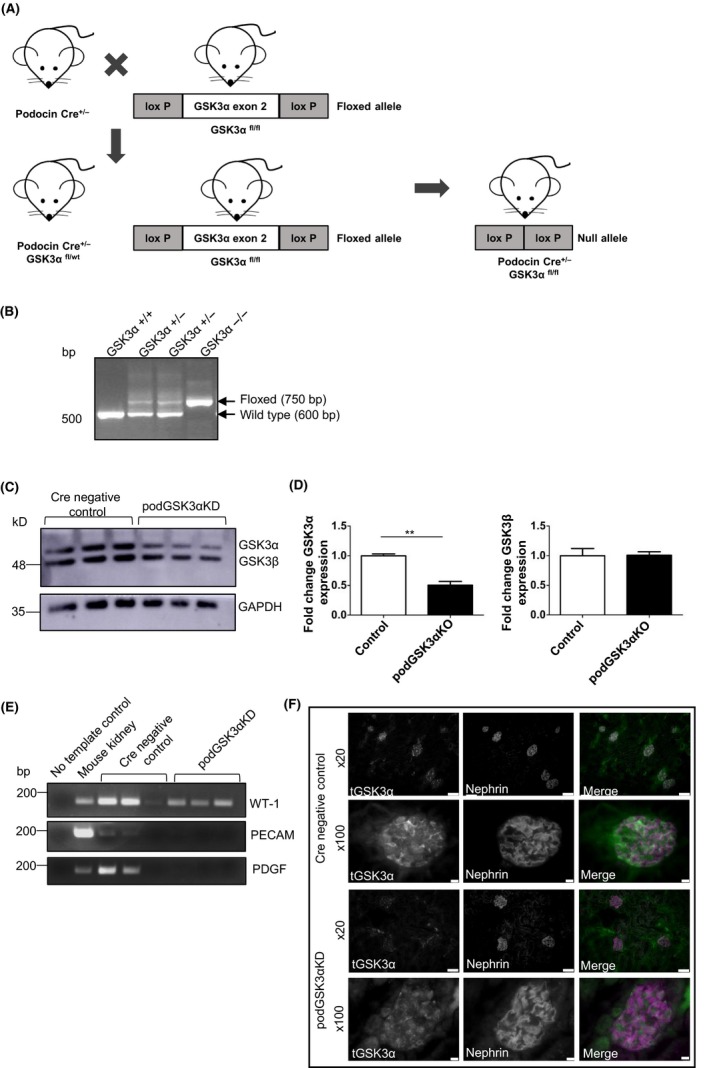
Generation of podocyte‐specific GSK3α knockdown mice. A, Breeding strategy used to generate podGSK3αKD mouse model showing deletion of GSK3α exon 2 in podGSK3αKD mice. B, Gel of end point polymerase chain reaction (PCR) products used to identify genotypes of podGSK3α mice. C, Western blot of podocyte primary culture cells from podGSK3αKD and littermate control mice. D, Densitometry of blot showing fold change of GSK3α and GSK3β levels normalized to GAPDH expression. Unpaired *t* test, **P < 0.01. podGSK3αKD podocytes have reduced expression of GSK3α. All primary podocytes express the podocyte marker WT‐1. E, Gel of end point PCR analysis of cDNA derived from podGSK3αKD and littermate control mice with WT‐1, PECAM and PDGF primers. Mouse kidney cDNA was used as a positive control. F, Immunofluorescent analysis of total GSK3α in podGSK3αKD and Cre negative control mice including co‐staining with the podocyte marker protein nephrin. Scale bar = 50 μm (×20); 75 μm (×100)

To study the effect of GSK3α deletion in podocytes we generated transgenic mice by crossing GSK3α floxed mice with mice expressing Cre recombinase under the control of a podocin promotor to confer podocyte specificity (Figure 2A). Genotypes of the progeny were confirmed using polymerase chain reaction (PCR) (Figure 2B). Primary culture podocytes were also generated from podGSK3αKD mice and Cre negative control littermates and knockdown of GSK3α determined using Western blotting (Figure 2C, D). GSK3α expression was reduced by 50% in podGSK3αKD mice while GSK3β expression remained unchanged. We analyzed the purity of our primary cultures using sensitive PCR analysis of cDNA derived from the primary cultures. This revealed some expression of PECAM and PDGF (markers of glomerular endothelial and mesangial cells) in two of the Cre negative control samples, likely due to a small amount of glomerular contamination of the primary podocytes. However, in the GSK3αKD cells the cell population appeared to be predominantly of podocyte origin (Figure 2E). Finally, podocyte knockdown of GSK3α was confirmed by co‐staining kidney sections from podGSK3αKD and Cre negative control mice with total GSK3α and nephrin antibodies (Figure 2F).

### 3.3 Podocyte‐specific knockdown of GSK3α does not ameliorate the progression of diabetic nephropathy in an STZ model of diabetes

**Figure 3:**
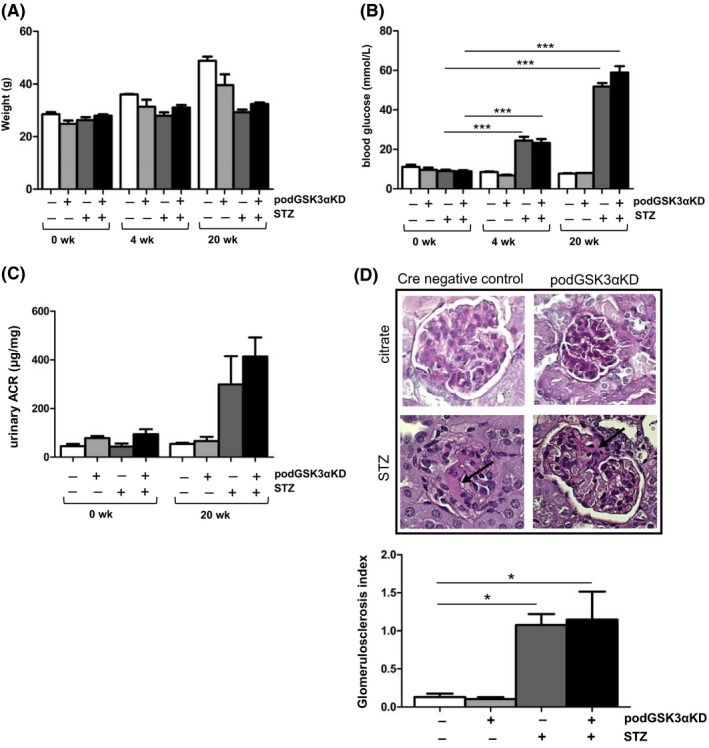
Podocyte‐specific knockdown of GSK3α does not ameliorate the progression of diabetic nephropathy in an streptozotocin (STZ) model of diabetes. A, Body weights in STZ‐treated (+STZ) and citrate buffer‐injected (−STZ) podGSK3αKD and control mice. One way ANOVA not significant, control mice n = 7, podGSK3αKD mice n = 9, citrate controls n = 3 for each genotype. B, Blood glucose measurements showing successful induction of STZ‐induced diabetes at 4 wk. STZ treated (+STZ); citrate controls (−STZ). One way ANOVA, ***P < 0.001. C, Urinary albumin:creatinine in STZ‐treated (+STZ) and citrate buffer‐injected (−STZ) mice at 20 wk. One way ANOVA not significant, control mice n = 7, podGSK3αKD mice n = 9, citrate controls n = 3 for each genotype. D, Representative periodic acid Schiff staining and glomerulosclerosis index of STZ‐treated mice (+STZ) and citrate controls (−STZ) at 20 wk. Mild glomerulosclerosis observed in STZ‐treated animals (arrowed) but no difference between podGSK3αKD mice controls, one way ANOVA, *P < 0.05, >30 glomeruli per mouse analyzed, three mice per group.

Previous studies have shown improved insulin sensitivity and glucose handling in skeletal muscle cells of total body GSK3α knockout mice.^5^ Since podocytes are insulin responsive cells,^29^ we sought to determine whether podocyte‐specific deletion of GSK3α would ameliorate the progression of diabetic nephropathy in a mouse model of diabetes by improving insulin sensitivity. Diabetes was induced in podGSK3αKD mice and Cre negative control littermates by STZ administration at 6 weeks of age. Successful induction of diabetes was confirmed by measuring blood glucose at 4 weeks and hyperglycemia as well as body weight was identical in knockdown and control groups (Figure 3A,B). Urinary albumin creatinine ratio (uACR) was increased at 20 weeks in both knockdown and control groups after STZ induction with evidence of mild glomerulosclerosis on histological examination, but no statistical differences were observed between them (Figure 3C,D) showing that the progression of diabetic nephropathy was not attenuated by podocyte GSK3α knockdown.

### 3.4 Knockdown of GSK3α does not improve insulin sensitivity with respect to PI3 kinase signaling

**Figure 4:**
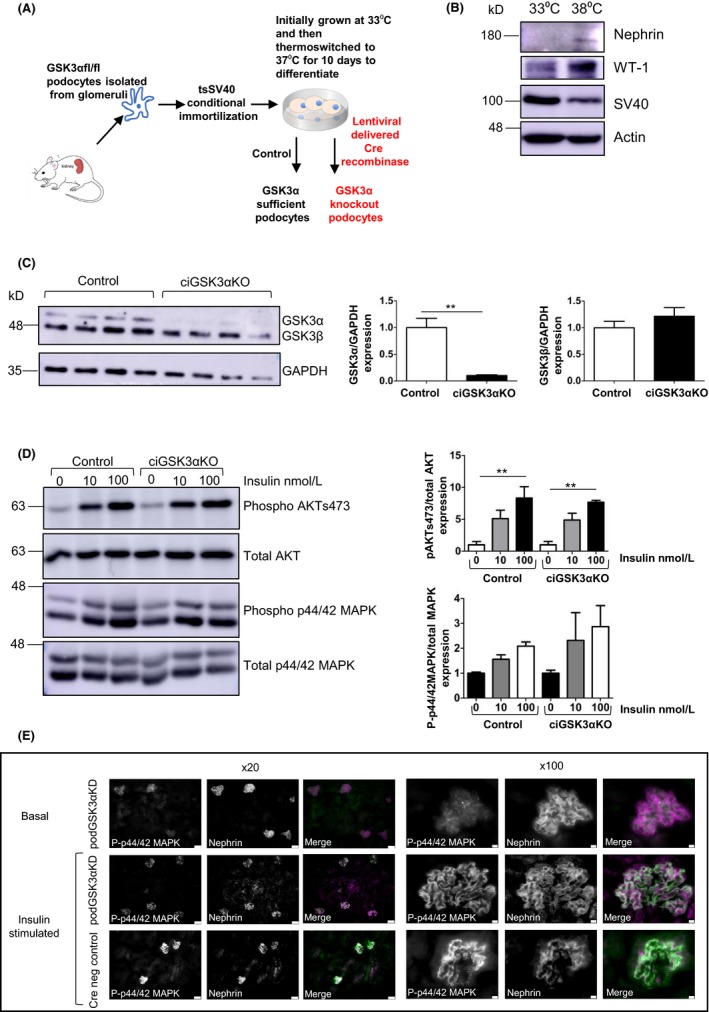
Knockdown of GSK3α does not improve insulin sensitivity with respect to PI3 kinase signalling. A, Development of ciGSK3αKO cells. Isolation of primary podocytes, conditional immortalization using SV40 construct and excision of GSK3α using lentiviral‐delivered Cre recombinase. B, ciGSK3αKO cells express markers of differentiated podocytes after thermoswitching. C, Western blot and densitometry of ciGSK3αKO cells showing >95% knockdown of GSK3α. Unpaired *t* test, **P < 0.01. D, Western blot and densitometry of ciGSK3αKO and control cells stimulated with 10 and 100 nmol/L insulin. No differences in phosphorylated AKT (pAKT) serine 473 or phosphorylated 44/42MAPK (p44/42MAPK) between the two groups. ANOVA with Tukey's post hoc test, **P < 0.01. n = 3 experiments. E, Immunofluorescent analysis of p44/42 MAPK performed in podGSK3αKD and Cre negative control mice that were fasted overnight and given insulin (6 nmol/L) for 10 min. Podocyte‐specific phosphorylation of MAPK is shown by co‐localization with nephrin (shown in white in merged images). Scale bar = 50 μm (×20); 75 μm (×100). MAPK, mitogen‐activated protein kinase

Podocyte GSK3α knockdown did not have a protective effect on the progression of STZ‐induced diabetic nephropathy but it is possible that this result was due to incomplete knockdown of GSK3α in our transgenic mouse model. To determine whether an increased level of GSK3α knockdown would be more effective in enhancing insulin sensitivity at a cellular/molecular level, we developed a temperature‐sensitive conditionally immortalized cell line from podocytes isolated from GSK3α floxed mice (Figure 4A). This cell line switched off the SV40 transgene following differentiation at the non‐permissive temperature of 38°C and expressed typical podocyte marker proteins (Figure 4B). Using this in vitro system, we were able to generate podocytes with >95% knockdown of GSK3α (Figure 4C). Both conditionally immortalized GSK3α knockout podocytes (ciGSK3αKO) and non‐transduced control cells responded to insulin stimulation, significantly increasing phosphorylation of AKT at serine 473 and exhibiting similar levels of glucose uptake (Data not shown). However, there was no difference between control and knockout cells indicating that even near‐total knockdown of GSK3α expression did not improve podocyte insulin sensitivity (Figure 4D).

We also studied the dynamic regulation of mitogen‐activated protein kinase (MAPK) ex‐vivo in the podocytes of podGSK3αKD and Cre negative control mice challenged with insulin after an overnight fast (Figure 4E). This confirmed that podGSK3αKD mice were able to phosphorylate MAPK in response to insulin but there did not appear to be any enhancement of this phosphorylation compared with podocyte GSK3α sufficient animals.

### 3.5 Podocyte‐specific knockdown of GSK3α is not protective in in vivo and in vitro Adriamycin injury models

**Figure 5:**
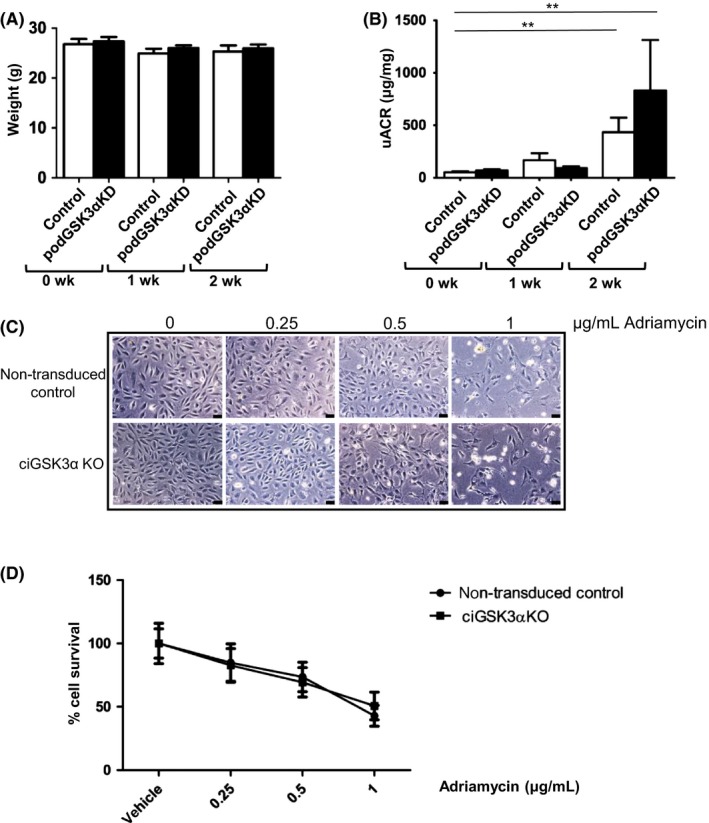
Podocyte‐specific knockout of GSK3α is not protective in in vivo and in vitro Adriamycin injury models. A, Body weights of Adriamycin‐treated mice. (B) Urinary albumin:creatinine in Adriamycin‐treated mice showing increased albuminuria in both podGSK3αKD and control mice at 1 and 2 wk. *t* test **P < 0.01. C, Representative images of ciGSK3αKO and non‐transduced control cells treated with Adriamycin for 24 h. D, Cell survival is identical in ciGSK3αKO and control cells treated with Adriamycin for 24 h. n = 3 experiments

In light of previous studies showing the beneficial effects of both pharmacological inhibition of GSK3 and podocyte‐specific genetic GSK3β deletion in doxorubicin injury models,^14^, ^15^, ^16^ we hypothesized that isoform‐specific knockdown of GSK3α in podocytes might have similar protective effects. Adriamycin nephropathy, a model of podocyte injury, glomerulosclerosis and fibrosis 30 was induced in podGSK3αKD mice by a single tail vein injection at 12 mg/kg body weight when mice were 8‐10 weeks of age and mice were monitored for 2 weeks before sacrifice. uACR was significantly increased at 2 weeks but no differences in albuminuria or body weight were apparent between podGSK3αKD mice and control littermates (Figure 5A,B).

To determine whether the increased GSK3α knockdown achieved in ciGSK3αKO cells would improve Adriamycin podocyte injury in vitro, we incubated cells with Adriamycin at 0, 0.25, 0.5 and 1 μg/mL for 24 hours. Both ciGSK3αKO and control cells looked unhealthy and were dying at an Adriamycin dose of 1 μg/mL (Figure 5C). Determination of cell number at Adriamycin concentrations of 0‐1 μg/mL using the non‐biased IN CELL computer‐based analyzer, confirmed that GSK3α deletion had no beneficial impact on cell survival (Figure 5D).

### 3.6 Podocyte GSK3α knockdown is detrimental causing increased uACR in vivo and increased cell death with impaired autophagic flux in ciGSK3αKO cells

**Figure 6:**
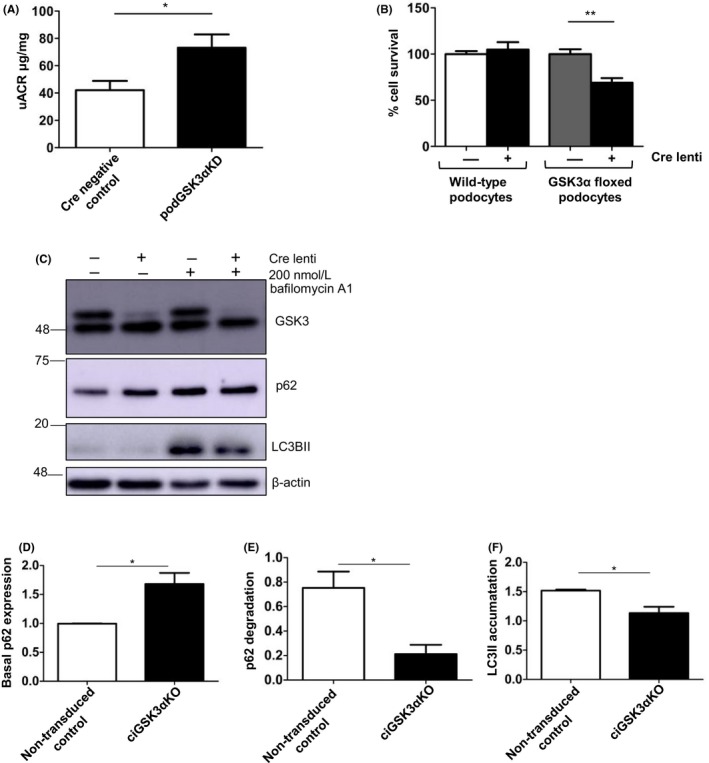
Podocyte GSK3α knockdown is detrimental causing increased urinary albumin creatinine ratio in vivo and increased cell death with impaired autophagic flux in ciGSK3αKO cells. A, Urinary albumin:creatinine is increased in podGSK3αKD mice. Unpaired *t* test, *P < 0.05. n = 12‐13 mice per group. B, Cell survival assay showing 25% cell death in ciGSK3αKO podocytes 7 d after transduction when compared with untransduced control cells or Cre lenti transduced wild‐type podocytes. ANOVA with Tukey's post hoc test, **P < 0.01. wild‐type cells, n = 3, GSK3α floxed cells n = 6 experiments. C, Representative blots of ciGSK3αKO and control cells treated with bafilomycin A1 for 4 h. D, Densitometry showing increased basal expression of p62 in ciGSK3αKO cells. Unpaired *t* test, *P < 0.05 n = 3. E, Level of p62 degradation represented by the difference in p62 levels ± bafilomycin A1 in ciGSK3αKO and control cells. Unpaired *t* test, *P < 0.05 n = 4. F, Accumulation of LC3II represented by the difference in LC3II levels ± bafilomycin A1 in ciGSK3αKO and control cells. Unpaired *t* test, *P < 0.05 n = 3

Although podGSK3αKD mice did not exhibit any overt phenotypic differences to controls with regard to body weight and kidney histology (Figure S2A,C), we observed a small but significantly increased basal uACR in podGSK3αKD mice at 6 weeks of age (Figure 6A; Figure S2B). We sought to further investigate the apparent detrimental effect of GSK3α reduction in podocytes by performing in vitro studies on ciGSK3αKO cells. Although we did not see any evidence of podocyte loss in our mouse model (Figure S2D), a cell survival assay performed 7 days after gene knockout using our in vitro model showed a 25% rate of cell death in ciGSK3αKO cells when compared to controls (Figure 6B). GSK3α has been found to be a critical regulator of autophagy, and its loss in a mouse global knockout model results in accelerated senescence in multiple tissues when aged.^31^ Previous studies have shown the importance of autophagy for podocyte homeostasis^32^, ^33^, ^34^, ^35^, ^36^ so we assessed autophagy in ciGSK3αKO cells. We observed that the polyubiquitin‐binding protein p62, which is degraded by autophagy, was increased in ciGSK3αKO cells (Figure 6C,D). However, although a higher basal level of p62 is suggestive of reduced autophagy in ciGSK3αKO cells, autophagy is a dynamic process and should be measured as a flux event. This can be represented by the difference in the amount of LC3II (which correlates with autophagosome number) in ciGSK3αKO and control cells in the presence or absence of the lysosomal inhibitor bafilomycin A1.^19^ We did not observe a clear band corresponding to LC3I but it is known that the sensitivity of immunoblotting for LC3I is much lower than for LC3II and that differences in LC3II accumulation between samples is the most accurate way to assess autophagy by Western blot.^19^ Differences in the levels of p62 before and after bafilomycin exposure indicate the amount of p62 that would normally be degraded by autophagy in each cell type. Using this approach, we found that the levels of p62 degradation (Figure 6E) and LC3II accumulation (Figure 6F) were significantly reduced in ciGSK3αKO cells indicative of impaired autophagic flux. We also assessed if enhancing autophagy in ciGSK3αKO cells by co‐incubating with the mTOR inhibitor Rapamycin would affect cell survival but did not find this to be the case (Figure S3).

## 4 DISCUSSION

We show here that in contrast to previous research indicating that podocyte‐specific genetic knockout of GSK3β has a beneficial effect in models of kidney disease; podocyte ablation of GSK3α is not similarly protective. Our findings indicate that conversely, its loss is mildly detrimental revealing a role for the α isoform in podocyte homeostasis.

Mice with global knockout of GSK3α have been shown to have enhanced insulin sensitivity^5^ albeit in a strain‐dependent manner.^8^ We initially hypothesized that genetic knockdown of podocyte GSK3α might attenuate albuminuria in an STZ‐induced model of diabetes due to increased podocyte insulin sensitivity. However, this was not the case.

In our animal model podocyte GSK3α expression was reduced by only approximately 50%, which we think was because our podocin Cre driver lost some of its Cre recombinase potency as a result of epigenetic silencing due to a high frequency of CpG dinucleotides.^37^ It is therefore possible that our in vivo results were due to incomplete gene knockdown. However it is important to remember that pharmacological GSK3 inhibitors cause a reduction in activity of approximately 25%^7^ which suggests that our findings are clinically relevant. The limited level of GSK3α knockdown in vivo led us to develop ciGSK3αKO podocytes with >95% gene knockout. However, we found that even this near complete knockdown of GSK3α in cultured podocytes did not elicit any increase in insulin sensitivity, consistent with our in vivo observations. Similarly, we did not observe any beneficial effect of podocyte GSK3α knockdown in in vivo and in vitro Adriamycin injury models contrary to results reported using podocyte‐specific GSK3β knockout mice.^13^, ^14^, ^38^ Our results suggest that GSK3α does not represent a potential target for augmentation of podocyte insulin signaling and that it is the reduction in GSK3β and not GSK3α activity that is responsible for the beneficial anti‐albuminuric and podocyte protective effects of pharmacological GSK3 inhibitors.^13^, ^15^, ^16^, ^39^ We have recently shown that some GSK3 enzyme activity is critically important for podocyte and kidney function both developmentally and in maturity. When both mammalian GSK3 isoforms (α and β) are lost genetically, or sufficiently suppressed pharmacologically, it causes a severe phenotype.^10^ This is also the case when the GSK3 gene is lost in similar cells in other species including the nephrocyte of *Drosophila*.^10^

Because GSK3 inhibitors are not isoform selective, future work to elucidate the beneficial and detrimental signaling pathways differentially regulated by GSK3α and β may facilitate the identification of new drug targets for the treatment of albuminuric kidney disease lacking the deleterious side effects that result from excessive suppression of GSK3 activity.

Interestingly, we noted a mild but significant increase in albumniuria in podGSK3αKD mice and further investigated this apparent detrimental effect in vitro using ciGSK3αKO cells. We observed that 7 days after near complete knockout of GSK3α, podocytes were more susceptible to cell death providing further evidence that GSK3α has a role in podocyte homeostasis. Although we did not find evidence of similar podocyte loss in podGSK3αKD mice, this may be due to the much lower degree of gene knockdown in vivo (approximately 50%) compared with the >95% GSK3α loss in ciGSK3αKO cells. Most GSK3 research has concentrated on the β isoform or the use of pharmacological GSK3 inhibitors and there are few reports in the literature describing the effects of specific loss of GSK3α. The original study describing the generation of whole body GSK3α knockout mice^5^ found that such mice were viable with no adverse phenotype, however subsequently it has been shown that male mice lacking GSK3α in their testicular germ cells are sterile.^40^ Additionally, a further recent elegant study of global GSK3α knockout mice has revealed that detrimental effects of GSK3α knockout become apparent as mice age. These mice had a reduced life span along with multiple age‐related pathologies attributable to a decreased level of autophagy.^31^ Autophagy, a conserved process that delivers proteins and damaged organelles to lysosomes for degradation and turnover^41^ has been shown to be important in podocyte homeostasis. In common with other terminally differentiated cell types, podocytes have a high level of autophagy under basal conditions^32^ and impairment of this process has been implicated in podocyte damage associated with aging,^35^, ^42^, ^43^ diabetic nephropathy,^34^ mitochondrial dysfunction and FSGS.^33^ Conversely, enhancement of autophagic activity has a protective effect in models of glomerular disease.^36^, ^44^ As global GSK3α knockout has been found to impair autophagy in multiple organ systems, we surmised that GSK3α knockout podocytes might also be autophagy deficient. We found this to be the case and that ciGSK3αKO podocytes had increased basal levels of p62 and reduced autophagic flux consistent with the cell death and increased albuminuria observed in our in vitro and in vivo podocyte GSK3α knockout models. We also assessed if impaired autophagy was directly linked to cell death in our knockout models by co‐incubating GSK3α knockout cells with the mTOR inhibitor Rapamycin but this did not have an effect in the short term. Going forward it will be of interest to significantly age the podocyte‐specific GSK3α mice, ideally using a Cre driver that is resistant to epigenetic degradation which has now been generated^45^ to assess for podocyte loss, glomerular disease and autophagocytic function. We speculate these would be abnormal.

In conclusion we have shown that partial or near‐total suppression of podocyte GSK3α is not protective in models of glomerular disease; its loss is, in fact, detrimental and is associated with impaired autophagic flux. This reveals a novel role for GSK3α in maintenance of podocyte function by promoting autophagy. Strategies to maintain or increase GSK3α activity may therefore represent a potential therapeutic target in albuminuric kidney disease.

## CONFLICT OF INTEREST

The authors declare no conflict of interest.

## AUTHOR CONTRIBUTIONS

Studies were conceived by JAH, RJC, JRW, GIW and SEQ. The majority of in vitro and in vivo work was performed by JAH with help from ACL and AFB. LN made the conditionally immortalized floxed GSK3alpha podocyte cell line.
