# Supplementary material for: Podocyte GSK3α is important for autophagy and its loss detrimental for glomerular function
Source: FASEB Bioadv. 2019 Jul 9;1(8):498–510. doi: 10.1096/fba.2019-00011 (PMC6902909; doi:10.1096/fba.2019-00011)
Supplement: Supplementary file 1 [file FBA2-1-498-s001.pdf]

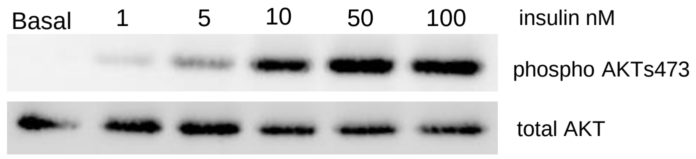

Supplementary Figure 1. Western blot of conditionally immortilised mouse podocytes stimulated with insulin at 1-100nM showing increased phosphorylation of AKT.

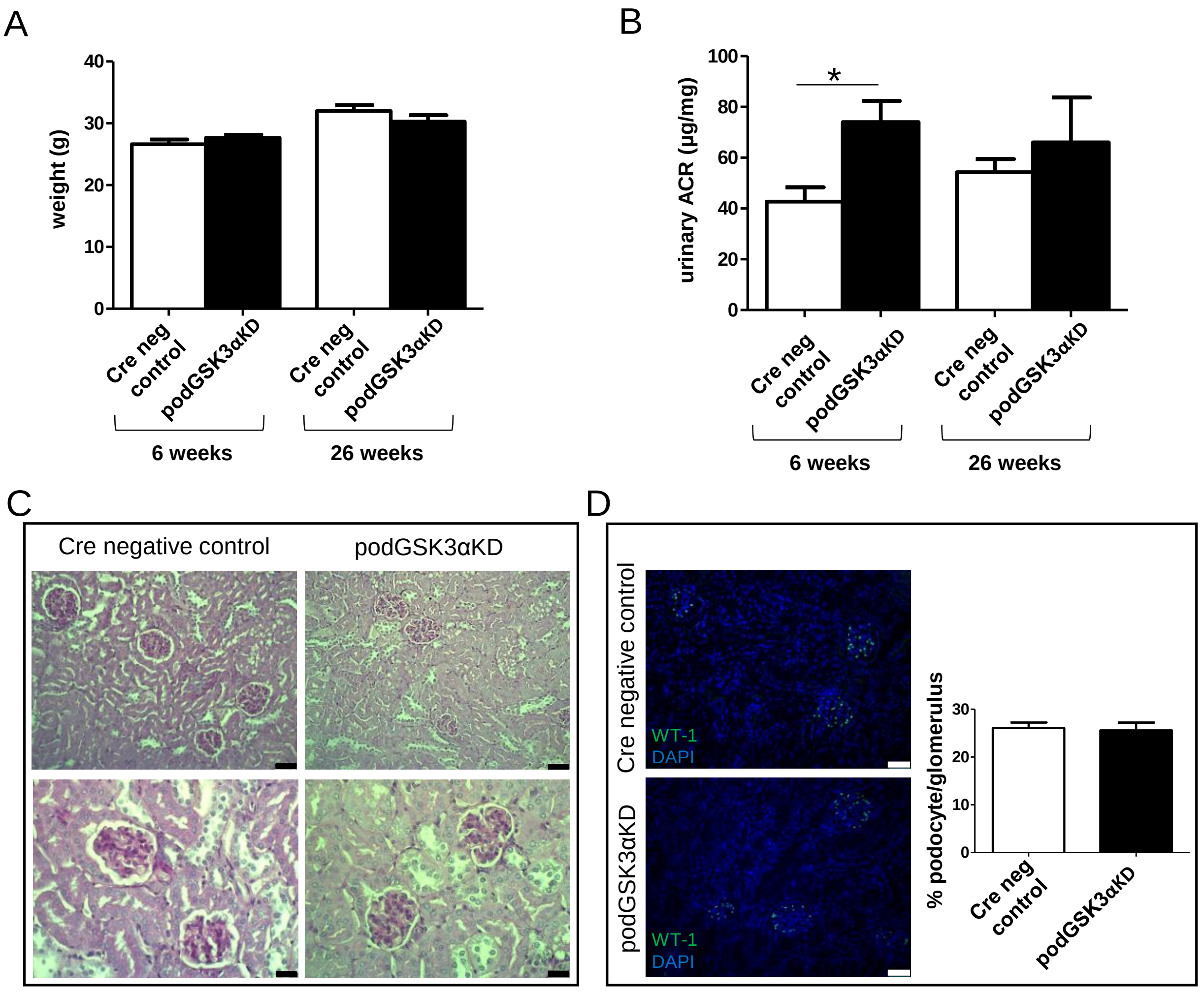

Supplementary Figure 2 Phenotype of pod GSK3αKD mice.

(A) Body weight of podGSK3αKD and littermate control mice at 6 and 26 weeks of age.

(B) Urinary albumin:creatinine in podGSK3αKD and littermate control mice at 6 and 26 weeks. Unpaired t test, *$p<0.05$. n=12-13 mice per group (6 weeks); n=3 per group (26 weeks).

(C) No histological differences apparent in PAS stained sections from podGSK3αKD and littermate control mice at 26 weeks. Scale bar=50μm (upper panel); 25μm (lower panel).

(D) WT-1 immunofluorescent staining of podGSK3αKD and Cre negative control littermate at 26 weeks. Nuclei counterstained with DAPI. Scale bar=50μm. No difference in % podocytes per glomerulus in podGSK3αKD mice compared with littermate controls. ≥8 glomeruli analysed per mouse, 3 mice per group, unpaired test.

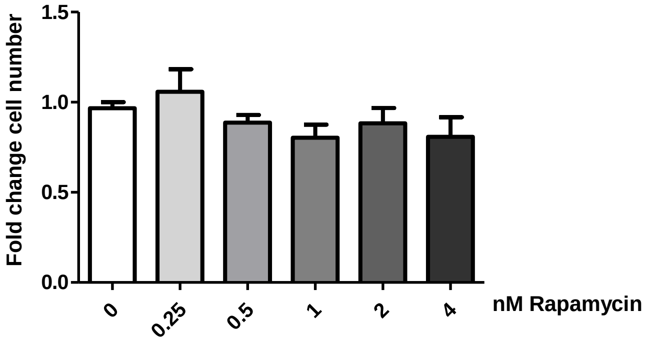

Supplementary Figure 3. Treatment of ciGSK3αKO cells with rapamycin at 0.25-4nM did not affect cell survival. GSK3α floxed podocytes were transduced with Cre expressing lentivirus for 24 hours to allow gene knockout, then treated with rapamycin at the above doses and cell number determined after 7 days. n=3 independent experiments.
